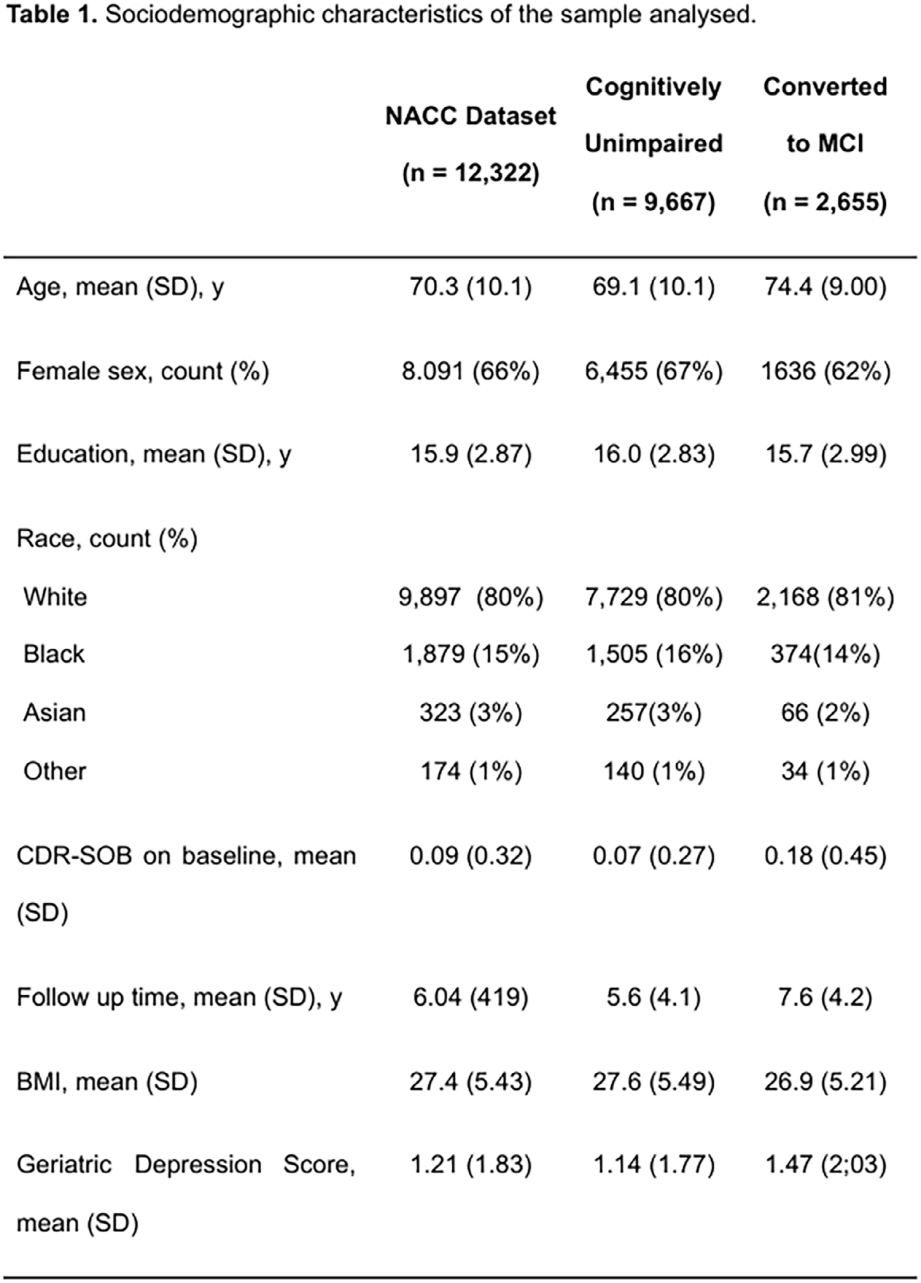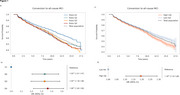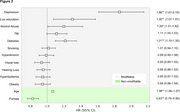# Stratification of Modifiable and Non‐Modifiable Risk Factors for Conversion to MCI in a Cognitively Unimpaired Cohort

**DOI:** 10.1002/alz70860_105892

**Published:** 2025-12-23

**Authors:** Daniel Arnold, João Pedro Ferrari‐Souza, Rodrigo C. Barros, Marco De Bastiani, Eduardo R. Zimmer, Wyllians Vendramini Borelli

**Affiliations:** ^1^ Universidade Federal do Rio Grande do Sul, Porto Alegre, Rio Grande do Sul, Brazil; ^2^ Universidade Federal do Rio Grande do Sul, Porto Alegre, RS, Brazil; ^3^ PUCRS, Porto Alegre, Rio Grande do Sul, Brazil; ^4^ Brain Institute of Rio Grande do Sul (InsCer), PUCRS, Porto Alegre, Rio Grande do Sul, Brazil; ^5^ McGill Centre for Studies in Aging, Montreal, QC, Canada; ^6^ Centro de Memória, Hospital Moinhos de Vento, Porto Alegre, RS, Brazil; ^7^ Clinical Hospital of Porto Alegre, Porto Alegre, Rio Grande do Sul, Brazil

## Abstract

**Background:**

Identifying risk factors for mild cognitive impairment (MCI) is crucial for developing effective prevention strategies. This study stratifies eleven modifiable and two non‐modifiable risk factors in a cognitively unimpaired (CU) population to predict the onset of MCI.

**Method:**

We analyzed data from the National Alzheimer's Coordinating Center (NACC) from 2005 to 2023 across 46 Alzheimer's Disease Research Centers (ADRCs). Eleven modifiable risk factors were evaluated: hearing loss, hypertension, BMI, depression, visual loss, education, hyperlipidemia, traumatic brain injury (TBI), alcohol abuse, smoking, and diabetes. Age and gender were considered non‐modifiable factors. Kaplan‐Meier survival analysis and Cox proportional hazards models were used for stratification. Modifiable risk factors were segmented into quartiles (Q1, Q2, Q3, Q4) and grouped into low‐risk (Q1, Q2) and high‐risk (Q3, Q4) to assess their association with MCI conversion. Demographic information is in Table 1.

**Result:**

The study included 12,322 CU individuals at baseline, with 2,655 converting to MCI. Survival analysis (Figure 1a) showed distinct differences in MCI conversion rates across quartiles. Participants in Q2, Q3, and Q4 had increased hazard ratios (HRs) for MCI onset compared to Q1 (Figure 1b). HRs for Q2 were 1.33 (95% CI: 1.19‐1.47), Q3 was 1.33 (95% CI: 1.19‐1.48), and Q4 was 1.36 (95% CI: 1.22‐1.52). The high‐risk group had a HR of 1.19 (95% CI: 1.10‐1.29) relative to the low‐risk group (Figure 1d). Key modifiable contributors included depression (HR 1.86; 95% CI: 1.61‐2.15), low education (HR 1.30; 95% CI: 1.07‐1.57), alcohol abuse (HR 1.20; 95% CI: 1.01‐1.42), and diabetes (HR 1.21; 95% CI: 1.10‐1.35) (Figure 2). Age (HR 1.06; 95% CI: 1.06‐1.07) increased risk, while female gender (HR 0.83; 95% CI: 0.76‐0.90) was protective.

**Conclusion:**

Stratification of risk factors reveals MCI risk heterogeneity linked to modifiable and non‐modifiable factors. Depression, low education, and diabetes were the most impactful for progression to MCI. Highlighting high‐risk individuals enables targeted interventions. Further research should focus on strategies addressing these modifiable risks to prevent MCI onset.